# Native Liquid Extraction Surface Analysis Mass Spectrometry: Analysis of Noncovalent Protein Complexes Directly from Dried Substrates

**DOI:** 10.1007/s13361-015-1152-8

**Published:** 2015-05-20

**Authors:** Nicholas J. Martin, Rian L. Griffiths, Rebecca L. Edwards, Helen J. Cooper

**Affiliations:** School of Biosciences, University of Birmingham, Edgbaston, Birmingham, B15 2TT UK; Department of Pharmacology, Vanderbilt University School of Medicine, Nashville, TN 37232 USA

**Keywords:** Native mass spectrometry, Proteins, Liquid extraction surface analysis, LESA, Liquid microjunction sampling, Direct surface sampling, Dried blood spots

## Abstract

Liquid extraction surface analysis (LESA) mass spectrometry is a promising tool for the analysis of intact proteins from biological substrates. Here, we demonstrate native LESA mass spectrometry of noncovalent protein complexes of myoglobin and hemoglobin from a range of surfaces. Holomyoglobin, in which apomyoglobin is noncovalently bound to the prosthetic heme group, was observed following LESA mass spectrometry of myoglobin dried onto glass and polyvinylidene fluoride surfaces. Tetrameric hemoglobin [(αβ)_2_^4H^] was observed following LESA mass spectrometry of hemoglobin dried onto glass and polyvinylidene fluoride (PVDF) surfaces, and from dried blood spots (DBS) on filter paper. Heme-bound dimers and monomers were also observed. The ‘contact’ LESA approach was particularly suitable for the analysis of hemoglobin tetramers from DBS.

**Graphical Abstract:**
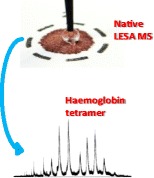
ᅟ

ᅟ

## Introduction

Soon after its introduction, it was demonstrated that electrospray ionization (ESI) [1] was capable of retaining specific noncovalent interactions [2]. Katta and Chait demonstrated ESI of holo-myoglobin (in which the apo-myoglobin is noncovalently bound to the prosthetic heme group) [3]. Light-Wahl et al. showed that it was possible to preserve noncovalent quaternary associations by ESI, detecting the intact tetramer of hemoglobin [4]. The literature now abounds with examples of ESI mass spectrometry of protein–metal, protein–ligand, protein–DNA, and protein–protein complexes, and this field of research has been dubbed native mass spectrometry [5]. Native mass spectrometry is able to define the stoichiometry of protein subunits in a multi-protein complex and requires a fraction of the sample or analysis time required for NMR or X-ray crystallography [6].

The conditions needed to retain a protein in its native (like) state are different from those used typically in ESI mass spectrometry. Buffers cannot contain organic solvent or have low pH. Such buffers unfold and protonate the protein, which does improve ionization efficiency but disrupts any noncovalent interactions [7]. In native mass spectrometry, proteins are electrosprayed from aqueous solutions containing volatile buffers such as ammonium acetate or triethylammonium bicarbonate at near neutral pH [8]. Consequently, only basic amino acid residues such as lysine will undergo reversible proton exchange with the solvent. Proton exchange takes place on residues at the surface of the protein and amino acid residues folded within the protein structure are shielded from protonation [9]. As a result, the charge states of the protein ions are generally lower than that of a typical denaturing ESI experiment [10], and time of flight mass spectrometers have become the instruments of choice because of their high upper mass-to-charge limit [11].

Other ionization techniques have been applied to the analysis of noncovalent protein complexes, including matrix-assisted laser desorption/ionization (MALDI) [12], and variants of ESI such as electrosonic spray ionization [13] and, more recently, paperspray ionization [14]. Here, we use liquid extraction surface analysis (LESA) mass spectrometry to probe noncovalent protein complexes directly from dried surfaces. LESA is a surface sampling technique that is often thought of as an ambient ionization technique alongside techniques such as desorption electrospray ionization (DESI). In fact, it is an ambient extraction process coupled with conventional ESI. LESA is a commercial variant of the liquid microjunction surface sampling probe (LMJ SSP) developed by Van Berkel [15] made available on the Triversa Nanomate nanoelectrospray platform [16]. The LESA extraction process is very simple: a droplet of solvent is applied to the surface of a substrate by a robotically controlled pipette tip. The droplet is held for a few seconds, forming a liquid microjunction between the pipette tip and the surface and allowing the passive diffusion of analyte molecules from the surface and into the solvent. The sample is then re-aspirated and infused into the mass spectrometer via ESI. The approach is suitable for a range of different analytes, including small molecules, lipids, proteolytic peptides, and intact proteins [17–20]. Intact proteins have been detected by LESA mass spectrometry from substrates, including dried blood spots (DBS) [16, 21], thin tissue sections [20, 22], and bacterial colonies growing on agar [23]. Nevertheless, prior to this work, LESA mass spectrometry of noncovalent protein complexes directly from dried substrates has not been demonstrated. The potential benefits of native LESA mass spectrometry over standard native ESI mass spectrometry are the ability to probe noncovalent protein interactions from biological substrates without the need for sample clean-up and, perhaps more importantly, to get direct insight into biological processes within that substrate. In principle, native LESA mass spectrometry could provide the ability to analyze protein complexes or drug–protein interactions directly from tissue or DBS. A potential drawback of native LESA mass spectrometry also arises from the lack of sample clean-up. In some cases, the complexity of the biological substrate may prevent ionization of intact protein complexes. (Nevertheless, it should be noted that an important feature of LESA is that the extraction and ionization events are decoupled, unlike other ambient techniques, providing opportunity to incorporate sample clean-up steps if necessary).

The challenge for native LESA mass spectrometry is that drying is known to denature proteins and disrupt noncovalent interactions [24]; however, for protein complexes to be observed by LESA MS, the noncovalent interactions must either survive the drying process or reform in the sampling process. Reports of noncovalent complex analysis by MALDI indicate that noncovalent interactions can survive crystallization within a MALDI matrix [12], so may survive drying on a surface. In this work, we demonstrate that LESA mass spectrometry can be successfully applied to the analysis of noncovalent protein complexes (holomyoglobin, hemoglobin dimers, and tetramers) directly from dried surfaces, including glass, polyvinylidene fluoride (PVDF), and DBS on filter paper. For DBS, potential challenges include drying and coagulation: blood clotting leads to the activation of platelets resulting in red blood cell membrane rupture and hemolysis, whilst drying red blood cells can lead to further lysis and auto oxidation of hemoglobin [25, 26]. Furthermore, blood has a high sodium content (between 135 and 145 mM) [27]. Despite these challenges, our results show that noncovalent hemoglobin tetramers can be sampled directly from DBS. The native LESA mass spectrometry approach may, therefore, be useful as a tool for the screening and diagnosis of hemoglobin synthesis disorders, in addition to the analysis of other noncovalent protein interactions.

## Materials and Methods

### Standard Proteins

Lyophilized horse cardiac muscle myoglobin and human hemoglobin were purchased from Sigma Aldrich (Gillingham, UK) and used without further purification. One hundred μM solutions of each were prepared in HPLC grade water, (J. T. Baker, Deventer, The Netherlands). Five μL aliquots were spotted onto Immobilon P PVDF membranes (Millipore, Watford, UK) and plain glass microscope slides (Thermo Scientific, Loughborough, UK) at marked positions. Glass microscope slides were first prepared by rinsing with HPLC grade water and air drying. Following deposition of protein solutions, samples were left to air dry for 4 h prior to analysis.

### Dried Blood Spots

The work was approved by the University of Birmingham STEM Ethical Review Committee (ERN_12-0782A and ERN_14-0454). Dried blood spots (DBS) were acquired from healthy human adult donors via finger prick onto NHS blood spot (Guthrie) cards, Ahlstrom grade 226 filter paper ID Biological Systems, (Greenville, SC, USA) and dried overnight prior to analysis.

### Surface Sampling

#### LESA Sampling

LESA was carried out by use of a Triversa Nanomate nanoelectrospray platform (Advion Biosciences, Ithaca, NY, USA). The dried sample substrates were mounted onto the LESA universal adaptor plate and an image was acquired using an Epson Perfection V300 photo scanner. The LESA Points software (Advion) was used to select the precise location of the substrate to be sampled. The universal adapter plate was placed in the Triversa Nanomate. Ten mM ammonium acetate (Fisher Scientific, Loughborough, UK), balanced to pH 6.8 with acetic acid (Sigma Aldrich, Gillingham, UK), was used as an extraction and ionization solvent for the standard proteins. The extraction/ionization solvent for the DBS was 95:5 ammonium acetate (10 mM, pH 6.8):methanol (HPLC grade, J. T. Baker, Deventer, The Netherlands). In the extraction process, 7 μL of solvent was aspirated from the solvent well. The robotic arm relocated to a position above the sample and descended to a height of either 1.6 mm (Orbitrap analyses) or 0.6 mm (Q-TOF analyses) above the surface. Four μL of solvent was dispensed onto the surface. After a delay of 3 s, 4.5 μL of sample was re-aspirated into the pipette tip. The dispense/re-aspirate process was repeated once before samples were infused into the mass spectrometer via chip-based electrospray ionization. Samples were infused with a gas pressure of 1.0 psi and an ionization voltage of 1.7 kV. In some cases, LESA sampling of DBS was characterized by collapse of the liquid microjunction. In that event, DBS were left to dry for approximately 5 min and the same areas were resampled using the same extraction parameters, but only allowing one dispense/re-aspiration cycle (to prevent repeated collapse).

#### ‘Contact’ LESA Sampling

‘Contact’ LESA [23], in which the pipette tip comes into contact with the surface rather than forming a liquid microjunction, of DBS was carried out using the Triversa Nanomate coupled with the Synapt G2S mass spectrometer (Waters, Manchester, UK). In this process, 10 μL of solvent (95:5 ammonium acetate (10 mM, pH 6.8):methanol) was aspirated from the solvent well and the robotic arm relocated to a position above the DBS. The tip descended to a depth such that it was in contact with the DBS. Once in contact, 4 μL of solvent was dispensed and held in contact for 10 s; 4.5 μL of solvent was re-aspirated and infused into the mass spectrometer at a gas pressure of 0.3 psi and 1.5 kV.

### Direct Infusion Electrospray

For comparison, direct infusion electrospray mass spectra of solution-phase samples of the standard proteins were acquired. Ten μM solutions of myoglobin or hemoglobin were prepared in ammonium acetate (10 mM, pH 6.8). Five μL aliquots were introduced into the mass spectrometer via the Triversa Nanomate in positive mode at a gas pressure of 1.0 psi and an ionization voltage of 1.7 kV.

### Mass Spectrometry

Mass spectrometry experiments were performed on an Orbitrap Velos ETD mass spectrometer (Thermo Fisher Scientific, Bremen, Germany) and a quadrupole time of flight Synapt G2S, (Waters, Manchester, UK).

For the Orbitrap Velos, all mass spectra were recorded in full scan mode in the Orbitrap at a resolution of 100,000 at *m/z* 400. Automatic gain control (AGC) target was 1 × 10^6^ charges with a maximum inject time of 1000 ms. The ion transfer tube was set at 250°C for all experiments other than LESA of dried blood spot samples, in which it was set at 200°C. The *m/z* range was 600–4000. Each scan comprised 50 co-added microscans. Data were acquired for 10 minutes. Data were analyzed using Xcalibur software (ver. 2.1; Thermo Fisher Scientific).

For the Synapt, all data were acquired in resolution mode without traveling wave ion mobility. For LESA experiments, the scan time was 2 s and *m/z* range was 1000–5000. For the ‘contact’ LESA experiments, the scan time was 10 s and the *m/z* range was 600–8000. The source temperature was set to 30°C and the cone voltage was set at 45 V. Data were acquired for 5 min in full scan mode only. Data were analyzed usinsg Mass Lynx software (ver. 4.1, Waters).

## Results and Discussion

The proteins studied in this work were myoglobin (Mb) and hemoglobin (Hb). In the native state, myoglobin comprises the polypeptide chain (apo-myoglobin) noncovalently bound to the prosthetic heme group. Hemoglobin exists as a noncovalently bound tetramer comprising two α-globin chains and two β-globin chains where each globin chain is noncovalently bound to a heme group. Assembly of the tetramer proceeds via association of two α^H^β^H^ dimers (^H^ indicates heme group) [28]. We have considered standard lyophilized proteins (Mb and Hb) and dried blood spots (Hb).

### Native LESA Mass Spectrometry of Myoglobin

**Figure 1:**
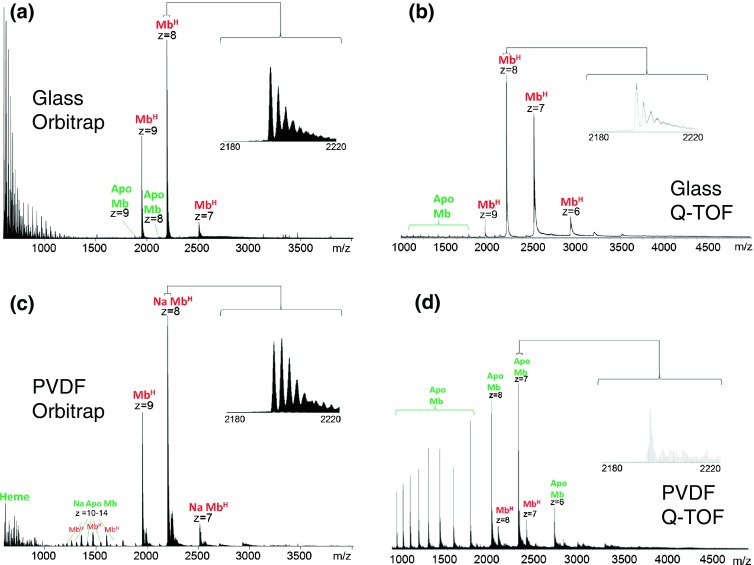
Native LESA mass spectra of myoglobin: (**a**) glass substrate, Orbitrap mass analyzer; (**b**) glass substrate, Q-TOF mass analyzer; (**c**) PVDF substrate, Orbitrap mass analyzer; (**d**) PVDF substrate, Q-TOF mass analyzer

**Figure 2:**
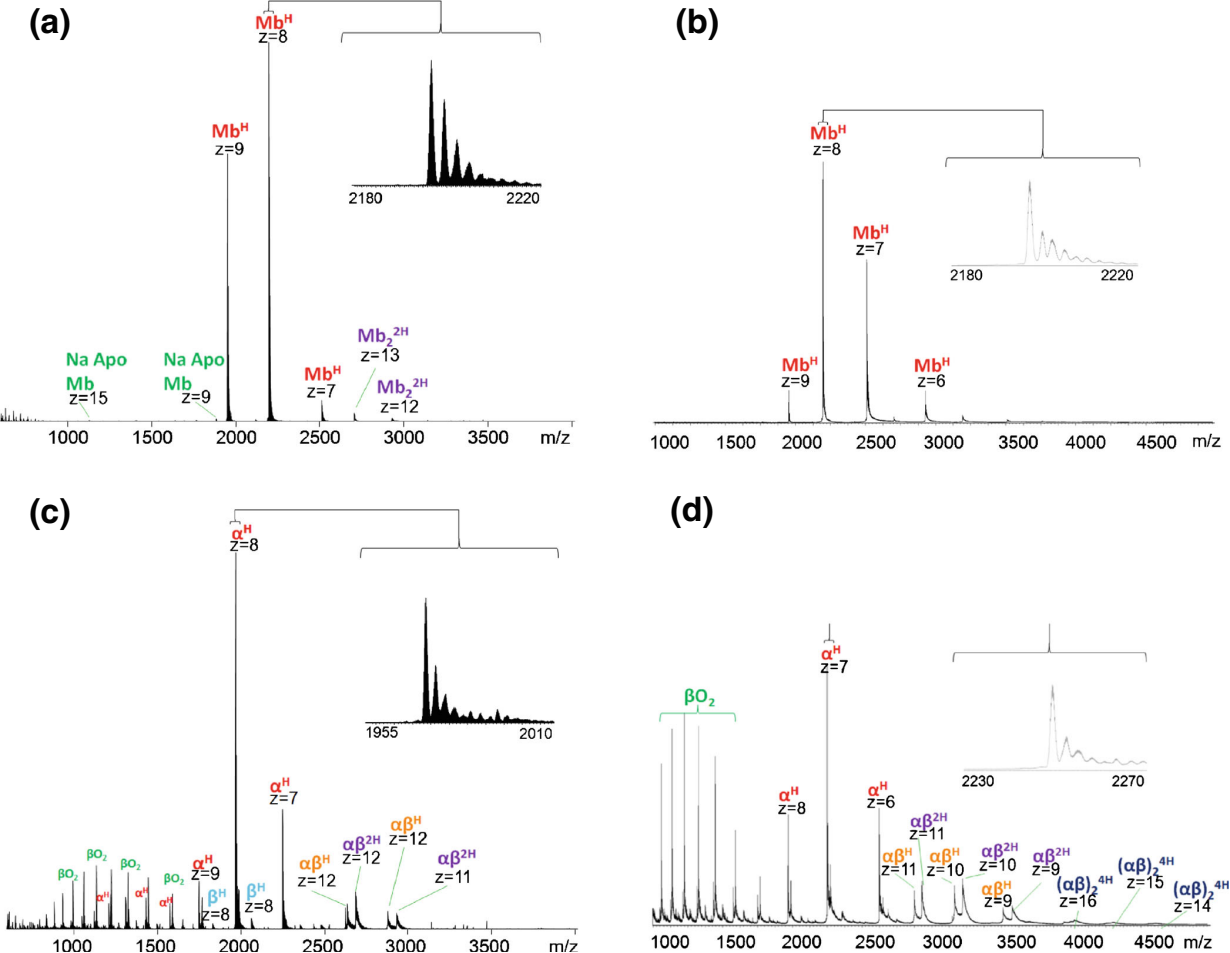
Direct infusion native ESI mass spectra of (**a**) myoglobin obtained with Orbitrap mass analyzer; (**b**) myoglobin obtained with Q-TOF mass analyzer; (**c**) hemoglobin obtained with Orbitrap mass analyzer; (**d**) hemoglobin obtained with Q-TOF mass analyzer

Figure 1 shows the results obtained following LESA mass spectrometry of myoglobin dried onto glass and PVDF surfaces. Figure 1a and c show results obtained on the Orbitrap and Figure 1b and d on the Q-TOF. LESA MS of myoglobin on glass resulted in detection of the noncovalent myoglobin–heme complex (holomyoglobin) Mb^H^. Dominant peaks in the Orbitrap mass spectrum correspond to the 9+, 8+, and 7+ charge states of holomyoglobin at *m/z* 1952.78, 2196.75, and 2510.42. (Stated *m/z* values from Orbitrap data are for the most abundant isotopic peak). The Q-TOF spectrum showed peaks corresponding to the 9+, 8+, 7+, and 6+ charge states of Mb^H^ at *m/z* 1953, 2197, 2510, and 2927. (Stated *m/z* values from Q-TOF data correspond to the maximum peak height). Differences in the observed charge states are likely due to the capillary temperatures of the Orbitrap and the Q-TOF: higher temperatures can favor the formation of higher charge states [29]. Higher temperatures can lead to greater unfolding of the protein, thus exposing more basic amino acid residues (with subsequent protonation). Additionally, higher temperatures may lead to more effective desolvation during ionization [10]. Heating during desolvation helps eliminate neutral molecules that become deposited on the protein, which may prevent it from obtaining high charge states [9, 29]. Peaks corresponding to apo-myoglobin were observed at low abundance in both the Q-TOF mass spectrum and the Orbitrap spectrum. Notably, the peaks corresponding to apomyoglobin are of higher abundance in the Q-TOF spectrum compared with those in the Orbitrap mass spectrum. Given that the samples were prepared identically and that higher temperatures were encountered in the Orbitrap (see above), it is likely that this observation is the result of differences in ion transfer parameters in the two instruments. There are numerous singly and doubly charged peaks below *m/z* 1200 in the Orbitrap mass spectrum, which correspond to a polymeric background ion with a repeating unit of mass 44 Da. For comparison, the direct infusion mass spectra of myoglobin obtained on the Orbitrap and Q-TOF instruments are shown in Figure 2a and b. The peaks corresponding to noncovalent complexes and the charge state distributions match those observed in the LESA mass spectra. There are no peaks corresponding to apomyoglobin, suggesting that dissociation occurs in the drying and/or extraction process. There are also no dominant peaks at *m/z* <1200, suggesting that those peaks observed in the LESA mass spectrum may originate from the glass substrate.

The dominant peak observed following LESA MS on the Orbitrap (Figure 1c) of myoglobin dried onto PVDF corresponded to the sodiated noncovalent complex in the 8+ charge state (*m/z* 2199.51). Sodiated Mb^H^ was also observed in the 7+ charge state (*m/z* 2513.58). Peaks corresponding to sodiated apomyoglobin in the +10 to +14 charge states were also observed. The LESA mass spectrum obtained on the Q-TOF (Figure 1d) was dominated by peaks corresponding to apomyoglobin in the +9 to +16 charge states. Relatively low abundance peaks corresponding to the +7 and +8 charge states of the noncovalent Mb^H^ complex (*m/z* 2510 and 2196) were observed. The results suggest that PVDF is not a suitable substrate for native LESA mass spectrometry. PVDF is a synthetic hydrophobic polymeric membrane. Hydrophobic surfaces have been postulated to denature proteins through interactions between hydrophobic amino residues and the surface. These interactions cause the protein to spread and unfold the native structure [30].

Mass spectra obtained from both instruments show evidence of salt adducts alongside the major protonated species. Salt adducts are a common problem for native mass spectrometry [31]. For solution-phase native mass spectrometry, some offline desalting procedures such as size exclusion chromatography and dialysis are available to prevent this occurrence [32]; however in the present case, there are very few options available as the LESA sampling routine cannot accommodate these offline desalting stages. Nevertheless, protonated ions remain the dominant species in all cases with the exception of the Orbitrap analysis of myoglobin from PVDF.

### Native LESA Mass Spectrometry of Hemoglobin from Glass and PVDF

**Figure 3:**
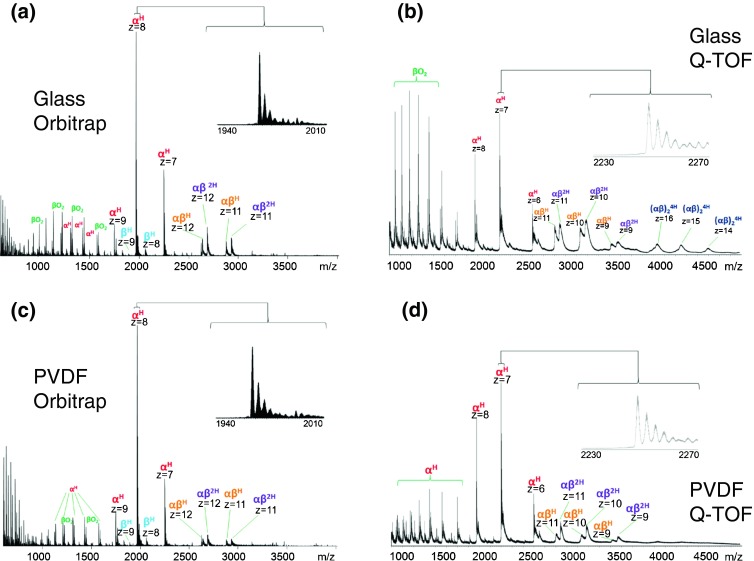
Native LESA mass spectra of hemoglobin: (**a**) glass substrate, Orbitrap mass analyzer; (**b**) glass substrate, Q-TOF mass analyzer; (**c**) PVDF substrate, Orbitrap mass analyzer; (**d**) PVDF substrate, Q-TOF mass analyzer

Figure 3 shows the results obtained following LESA MS of hemoglobin standard dried onto glass and PVDF substrates. For comparison, the direct infusion mass spectra of the protein are shown in Figure 2c and d. As described above, in its native state hemoglobin exists as a noncovalent tetramer of the form (αβ)_2_^4H^. Figure 3a shows the mass spectrum obtained on the Orbitrap following LESA of the glass substrate. The spectrum is characterized by noncovalent α^H^ and β^H^ monomers, noncovalent heterodimers (αβ)^2H^, and those with a single heme (αβ)^H^. Heme-deficient dimers have been reported in previous examples of native hemoglobin analysis [33, 34]. It is notable that the β^H^ monomer was of significantly lower abundance than the α^H^ monomer. Also observed are peaks corresponding to beta globin with a mass shift Δm +32 Da. This observation has been reported previously and has been speculated to be due to oxidation of the beta chain [33]. Tetramers were not observed. Similar results were obtained following LESA of the PVDF substrate (Figure 3c), although the relative abundance of the dimers was lower. The LESA MS results are in good agreement with the direct infusion ESI results (Figure 2). The relative abundance of the dimers was higher following direct infusion ESI and no dominant peaks at *m/z* <800 were observed.

Results obtained on the Q-TOF are shown in Figure 3b and d. LESA MS from the glass substrate resulted in detection of noncovalent heme-bound monomers, dimers (αβ)^2H^ and (αβ)^H^, and tetramers in the +16, +15, and +14 charge states (*m/z* 4070, 4345, and 4663, respectively). A similar mass spectrum was obtained following direct infusion ESI of hemoglobin (Figure 2d), although interestingly the relative abundance of the tetramers was greater in the LESA mass spectrum. The Q-TOF mass spectrum obtained following LESA of the PVDF substrate (Figure 3d) is similar to that obtained from glass in terms of species observed; however, the signal-to-noise was much lower. The tetramer peaks are barely discernible above the noise.

The tetramer peaks in these spectra are very broad ( width ~100 Th), which is consistent with previously published examples of native hemoglobin tetramers [14, 33]. This broadening is likely due to the presence of salt adducts and unevaporated solvent molecules. Commercially available time of flight mass analyzers do not have sufficient resolving power to distinguish monosodium adducts from their protonated counterparts at *m/z* >4000 [35], so the resulting peaks appear to be very broad, and exact masses cannot be determined. Dimer peaks observed following LESA from glass are much broader in the Q-TOF data than those in the Orbitrap data. It is clear from the Orbitrap data that for dimers, the protonated species are dominant, with sodiated and potassiated adducts also observed; however, it is not possible to distinguish between the various adducts in the Q-TOF mass spectra. A further contributor to peak width may be insufficient desolvation. The temperature of the Q-TOF inlet was 30°C as opposed to 250°C for the Orbitrap. Lower temperatures can prevent dissociation of unstable complexes (which could explain why it is possible to observe hemoglobin tetramers on the Q-TOF and not the Orbitrap); however, the complex may be associated with solvent adducts, thus generating broader peaks [36, 37].

### Native LESA Mass Spectrometry of Dried Blood Spots

**Figure 4:**
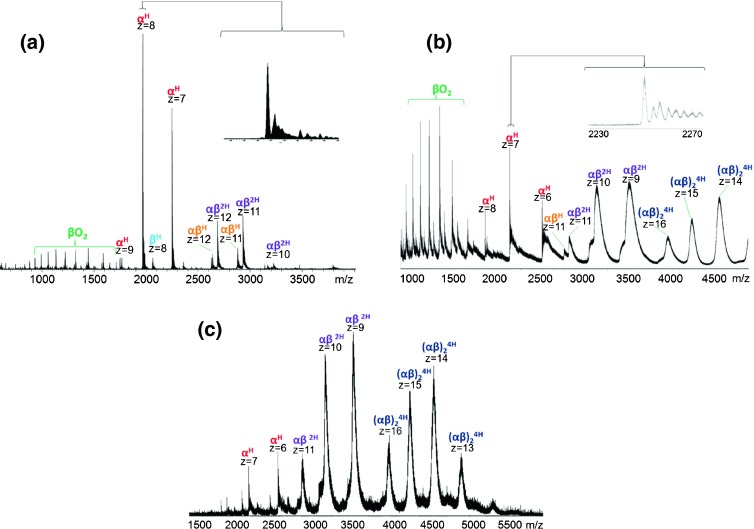
Native LESA mass spectra of hemoglobin from dried blood spots: (**a**) Orbitrap mass analyzer; (**b**) Q-TOF mass analyzer; (**c**) ‘contact’ LESA with Q-TOF mass analyzer

Figure 4 shows the results obtained following native LESA MS of dried blood spots on filter card. The concentration of hemoglobin in human blood is approximately 130 mg/mL for a healthy adult male and 120 mg/mL for a healthy adult female [38]. Results obtained from the Orbitrap (Figure 4a) were similar to those of the hemoglobin standard, showing the presence of (αβ)^2H^ dimers, (αβ)^H^ dimers, α^H^, β^H^, and unbound monomers. The relative abundance of the (αβ)^2H^ dimer was greater from the DBS sample than the protein standard. This observation may be the result of reducing the inlet capillary temperature to 200°C. Tetramers were not observed in the Orbitrap data; however, these were observed in the Q-TOF mass spectrum (Figure 4b). Native LESA MS using the Q-TOF resulted in detection of (αβ)_2_^4H^ tetramers, αβ^2H^ dimers, αβ^H^ dimers, α^H^, and β^H^. As for the standard protein, the tetramer peaks are broad (~100 Th) wide, but the relative abundance of the tetramers is much higher. Figure 4c shows the mass spectrum obtained following ‘contact’ LESA sampling [23] of DBS. The results suggest that this approach is particularly suitable for the analysis of Hb tetramers from DBS. The relative abundance of the peaks corresponding to the tetramers is greater than following standard LESA. In addition, the peak widths are ~80 Th. The observation of Hb tetramers directly from dried blood spots is an exciting one: the native LESA mass spectrometry approach could be useful in studying disorders of hemoglobin synthesis such as thalassemia. These disorders can result in the production of unusual hemoglobin tetramers such as Hb Barts, a tetramer composed of four gamma chains, or HbH, a tetramer composed of four beta chains [39].

## Conclusions

We have demonstrated that LESA mass spectrometry can be successfully applied to the analysis of noncovalent complexes of proteins dried onto surfaces. Holomyoglobin could be detected from glass and PVDF substrates, as could heme-bound hemoglobin dimers and monomers. Generally, glass outperformed PVDF as a native LESA substrate. Hemoglobin tetramers could be detected from glass microscope slides and DBS. The latter is particularly significant as DBS are a convenient clinical sampling format but present a highly complex sample. Results from ‘contact’ LESA of DBS were especially encouraging as the relative abundance of the tetramers was greater than observed via standard LESA. The Q-TOF mass spectrometer proved to be more successful than the Orbitrap when analyzing hemoglobin tetramers; however, the analysis of smaller complexes benefitted from the resolution provided by the Orbitrap.
